# AREDS Formula, Warfarin, and Bleeding: A Case Report from the Michigan Anticoagulation Quality Improvement Initiative

**DOI:** 10.1155/2014/754147

**Published:** 2014-08-28

**Authors:** Eric Puroll, Steven T. Heidt, Brian Haymart, James B. Froehlich, Eva Kline-Rogers, Geoffrey D. Barnes

**Affiliations:** University of Michigan Health System, Domino's Farms, Lobby A, MCORRP, 24 Frank Lloyd Wright Drive, Ann Arbor, MI 48106, USA

## Abstract

*Importance.* The anticoagulant warfarin has been shown to interact with other medications, vitamin K containing foods, and over-the-counter products. These interactions may inhibit or potentiate the effect of warfarin, resulting in serious clotting or bleeding events. *Observations.* We report the case of an 84-year-old woman with atrial fibrillation, prescribed warfarin in May 2010 for stroke prevention. Her international normalized ratio (INR) was stable until April 2013, when she was prescribed AREDS (Age Related Eye Disease Study) formula pills, an eye vitamin compound, to slow the progression of age-related macular degeneration. This change was not reported to the Anticoagulation Service. Eighteen days later, she presented to the ED with groin and back pain and an INR of 10.4. An abdominal CT revealed a retroperitoneal hemorrhage with extension in multiple muscles. Both warfarin and AREDS were discontinued and the patient was discharged to subacute rehabilitation. This case was reviewed by the Anticoagulation Service and actions were taken to prevent similar adverse events. *Conclusions.* This report provides an example of the potential danger of supplement use, in this case, AREDS formula, in patients prescribed warfarin, and the importance of communicating medication changes to the providers responsible for warfarin management.

## 1. Background

The anticoagulant warfarin is prescribed for a range of cardiovascular conditions, mostly for thromboprophylaxis. Although proven efficacious, warfarin is often tedious to manage, requiring frequent monitoring of the international normalized ratio (INR). It has been estimated that 10–16% of patients treated with warfarin will have a major bleeding event [1]. As a vitamin K antagonist, warfarin has been shown to interact with other medications, foods, and over-the-counter products [2]. These interactions may inhibit or potentiate the anticoagulant effect of warfarin, resulting in serious clotting or bleeding events. We report a case study involving an elderly female with atrial fibrillation who experienced spontaneous retroperitoneal bleeding after being prescribed an oral vitamin supplement for prevention of macular degeneration, AREDS (Age Related Eye Disease Study) formula, while taking warfarin.

## 2. Case Study

V.S. is an 84-year-old female with a history of paroxysmal atrial fibrillation, hypertension, hypothyroidism, glaucoma, cataract surgery, and right total hip arthroplasty. She began warfarin in May 2010 with a CHADS_2_ score of 2 and target INR range of 2.0 to 3.0. She was referred to the anticoagulation service at a large tertiary care center that manages yearly over 3,500 patients who are prescribed warfarin. The patient underwent scheduled INR testing per guidelines used by the service and continued treatment with warfarin as instructed, with fairly consistent INRs.

On March 20, 2013, she was referred to ophthalmology for complaints of “spots in vision.” Her vision changes were attributed to age-related macular degeneration and she was switched from lutein to AREDS formula, a vitamin supplement thought to slow progression of the disease [3]. The next day, she had her INR checked and received a letter 4 days later via standardized protocol notifying her that her INR was 1.9 and that she should remain on her current dose of warfarin. There was no mention of the AREDS formula in the documentation by the anticoagulation service and because the INR was only slightly below range, the next INR was scheduled for April 15, 2013 (~3 weeks later).

On the morning of April 8, 2013, she was seen for an urgent orthopedic visit after awakening with groin pain the previous night. V.S. described the pain as beginning the day before, while standing for a prolonged period. She was advised to use a walker, apply ice to the affected area, and use NSAIDs.

That evening, with progressively worsening pain, she presented to the Emergency Department with complaints of right groin and back pain. Physical exam revealed exquisite tenderness in her groin. An emergent abdominal CT scan revealed retroperitoneal hemorrhage with extension into multiple muscles.

**Figure 1:**
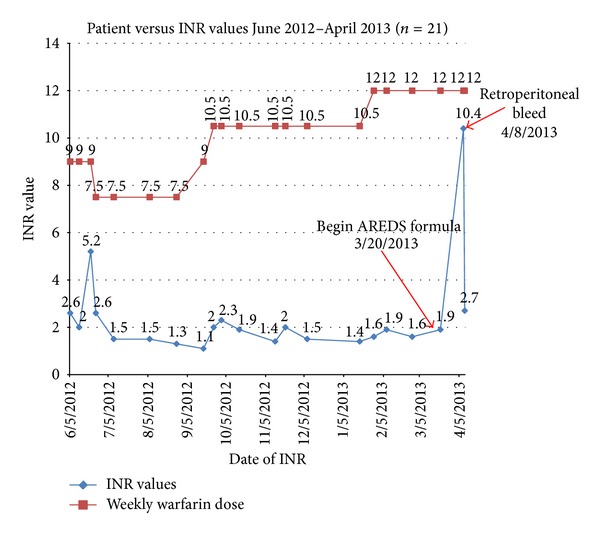
Timeline showing the patient's weekly warfarin dose (mgs), INR results, and events.

Her INR on admission to the ED was 10.4 (Figure 1). She was given 10 mgs of oral vitamin K, and she was admitted and transfused with 3 units of fresh frozen plasma and 2 units of packed red blood cells. Both warfarin and AREDS were discontinued and she was discharged four days later to a subacute rehabilitation facility.

## 3. Discussion

Supplement use in the United States is highly prevalent. Regular supplement use by the general population in the United States rose over the last twenty years, from 23.2% in 1987 to 49% in 2006 [4, 5]. Dietary supplements are used at an even greater level by the elderly American population, reported at 71% in 2005 [6]. Studies have shown that Americans generally use supplements to treat chronic problems, such as back pain, fatigue, anxiety, depression, headaches, and insomnia [7]. Those without chronic health issues state that they use dietary supplements for general health and well-being [8, 9]. Frequently, supplements/vitamins are perceived as being harmless, since they are available for purchase without a prescription.

The Age Related Eye Disease Study (AREDS) was an investigational study through the National Eye Institute which examined the use of eye antioxidants in the treatment of macular degeneration [3]. The study showed that patients with macular degeneration may slow the progression of the disease by taking an oral vitamin supplement containing copper; zinc; and high doses of vitamins A, C, and E, now referred to as AREDS formula (Table 1).

AREDS Report number 8 addressed the prevalence of adverse events within the study population [3]. In the report, possible adverse events are broken into two categories: those associated with antioxidants (including vitamin E) and those associated with zinc [3]. The AREDS investigators designated the primary adverse event cause as either a circulatory event or a skin/subcutaneous tissue event. The prevalence of adverse events among patients in the antioxidant group was 0.3% for circulatory events and 2.2% for skin/subcutaneous tissue events [3]. The prevalence of bleeding events was not reported explicitly in the study, although it should be noted that patients were warned about possible adverse effects from vitamin E, including an increased risk for hemorrhagic stroke [3].

Small clinical trials and case reports have offered conflicting opinions on the interaction of vitamin E and warfarin, although some have concluded vitamin E was responsible for the potentiation of warfarin's anticoagulant effect [10]. A case study reported a warfarin patient who initiated a regimen of 1200 IU daily vitamin E, which shortly led to the development of hematuria and ecchymoses, as well as an increased INR [11]. After stopping vitamin E and allowing his INR to return to therapeutic range, the patient initiated a regimen of 800 IU daily vitamin E, again leading to increased INR and ecchymoses [11]. Conversely, in a small trial of 12 patients taking between 100 and 400 IU of vitamin E alongside warfarin no patients experienced a clinical bleeding state [12]. A similar study of 12 patients on higher doses of vitamin E (1200 or 800 IU) showed similar results [13]. Although these small studies did not show significant adverse events, there still appears to be some patients who do have a reaction to vitamin E supplementation alongside warfarin anticoagulation. A proposed mechanism for this warfarin potentiation is direct inhibition of the vitamin K-dependent carboxylase by vitamin E quinone [14]. Regarding interaction between warfarin and AREDS supplementation, information is very limited, although one case report concluded AREDS vitamins were responsible for increased INR and extensive bruising [15].

V.S. reported no alteration in her medications or eating patterns. Having taken warfarin for two years prior to this event, she was familiar with the typical warfarin-associated precautions, including the need to be consistent with consumption of high vitamin K containing foods and notifying the anticoagulation services of medication changes. During a follow-up clinic visit, she noted that because she was prescribed “eye vitamins,” she did not feel the necessity to inform the service.

There was no documentation that the ophthalmologist acknowledged the potential interaction between AREDS and warfarin or educated V.S on the potential risks. Furthermore, there was no correspondence between the patient or ophthalmologist and the anticoagulation service regarding the prescription for AREDS.

Two months after this incident, this case was reviewed by a panel of clinicians from the anticoagulation service who meet regularly to discuss adverse events related to warfarin and determine the probable cause of the event. This team consists of the physician medical director, nurse administrator, a PharmD, and nurses involved with the patient cases being discussed. The panel found no other causes of the elevated INR besides the AREDS formula.

This and other cases of adverse events while on warfarin are routinely reviewed as part of a quality initiative within the Michigan Anticoagulation Quality Improvement Initiative (MAQI^2^), a consortium supported by Blue Cross Blue Shield of Michigan (BCBSM) involving seven organized anticoagulation services in Michigan. The Adverse Event Root Cause Analysis (ARC) is one example of several quality improvement strategies currently being conducted at the seven sites.

## 4. Process Changes

After the ARC Panel's review of the case, the following actions were taken to prevent similar adverse events.

- The MAQI^2^ Project Coordinator contacted the Department of Ophthalmology, including the Chief of the Retina Division and Chief of Clinical Practice to report the case.
- The patient visit summary, as part of the electronic medical record (EMR), was modified to inform patients taking warfarin to contact the anticoagulation service if they are prescribed AREDS formula.
- An alert was created and delivered to all clinical faculties in the Ophthalmology Department.
- All anticoagulation services participating in the MAQI^2^ consortium were notified of the adverse event and process changes made.

## 5. Conclusion

This case report provides an example of the potential danger of supplement use in patients taking warfarin and illustrates how these interactions may not be widely known by providers unfamiliar with anticoagulation. Furthermore, this case proves the importance of communicating medication changes, even for vitamin supplements, to the providers responsible for warfarin management.

As a result of this event, several important process changes were made, including changing the EMR to inform patients of possible interactions and informing the Ophthalmology Department of the adverse event. The careful review and changes to process of care for a large number of warfarin providers demonstrate the positive impact of participation in quality improvement registries.

## Figures and Tables

**Table 1:** AREDS formula daily dose (4 tablets).

Type of supplement	Amount	% Daily value
Vitamin A (100% as beta-carotene)	28,640 IU	573%
Vitamin C (ascorbic acid)	452 mg	753%
Vitamin E (dl-alpha-tocopheryl acetate)	400 IU	1333%
Zinc (zinc oxide)	69.6 mg	464%
Copper (cupric oxide)	1.6 mg	80%

Supplement information for Ocuvite Preservision AREDS formula tablets:

Bausch + Lomb Website. http://www.bausch.com/en/ECP/Our-Products/Eye-Vitamins/Age-Related-Eye-Vitamins-ECP/Preservision-Eye-Vitamins-ECP. Accessed 8/14/13.
